# Supplementary material for: A Novel POP-Ni Catalyst Derived from PBTP for Ambient Fixation of CO2 into Cyclic Carbonates
Source: Materials (Basel). 2023 Mar 7;16(6):2132. doi: 10.3390/ma16062132 (PMC10057775; doi:10.3390/ma16062132)
Supplement: Supplementary file 1 [file materials-16-02132-s001.zip › materials-2245437-supplementary.pdf]

materials

Supplementary Materials

A Novel POP-Ni Catalyst Derived from PBTP for Ambient Fixation of CO_2 into Cyclic Carbonates

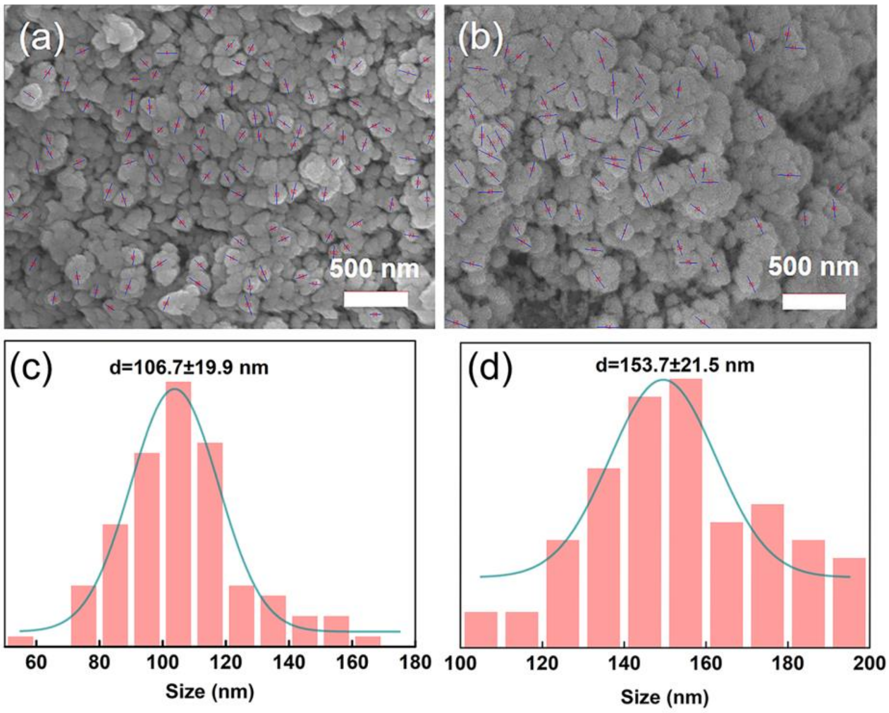

Figure S1. SEM image of PBTP (**a**) and POP-Ni (**b**); particle size distribution diagram of PBTP (**c**) and POP-Ni (**d**).

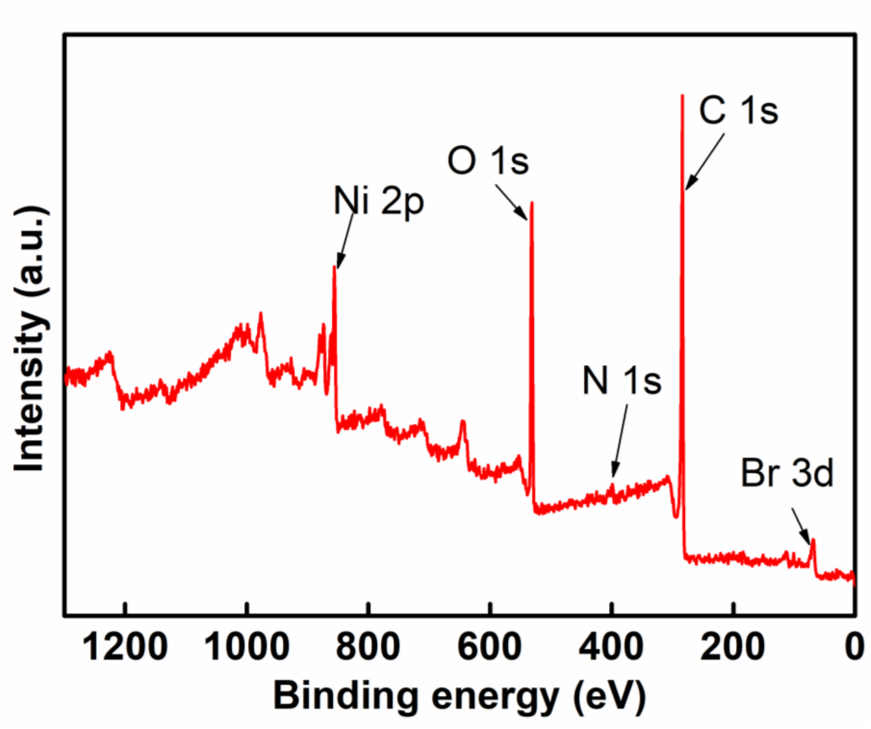

Figure S2. XPS spectrum of POP-Ni.

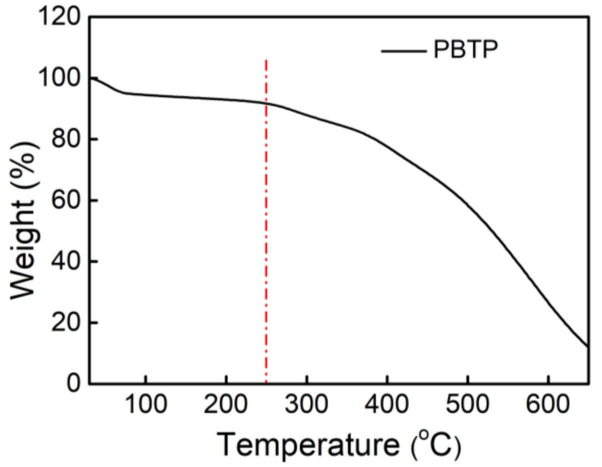

(**a**)

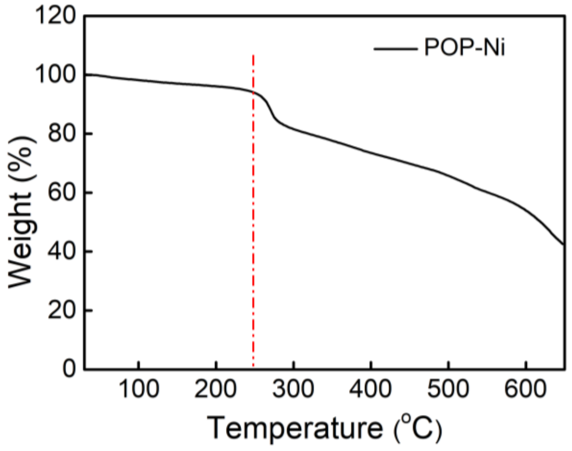

(**b**)

Figure S3. (a) TGA profile of PBTP; (b) TGA profile of POP-Ni.

Copyright: © 2023 by the authors. Licensee MDPI, Basel, Switzerland. This article is an open access article distributed under the terms and conditions of the Creative Commons Attribution (CC BY) license (https://creativecommons.org/licenses/by/4.0/).

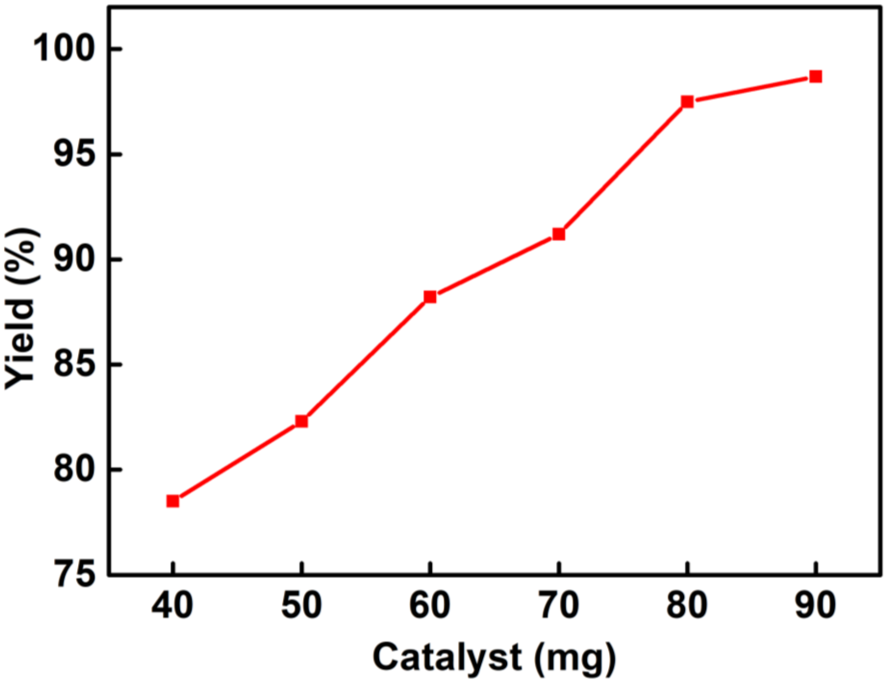

Figure S4. The catalytic activity corresponding to different catalyst amounts.

Table S1. Screening on the substrate scope for CO_2 cycloaddition with POP-Ni catalyst [a].

Entry	Epoxide	Product [b]	Major by-product [b]
1	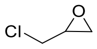	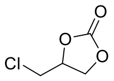	——
2	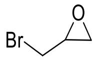	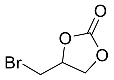	——
3	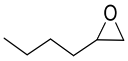	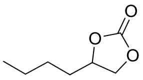	——
4	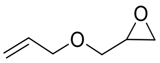	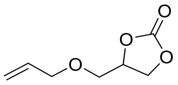	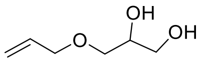
5	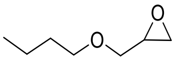	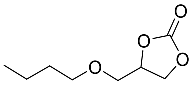	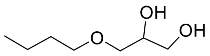
6	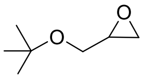	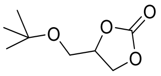	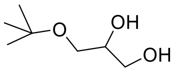
7	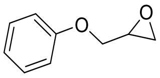	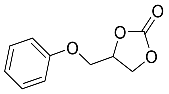	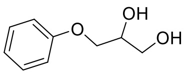

8	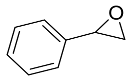	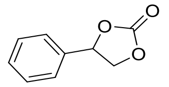	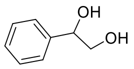

[a] Reaction condition：5 mmol of epoxide, 80 mg of POP-Ni, 0.5 mL of DMF, CO_2 (balloon), 80 °C and 24 h.
[b] Determined by GC-MS.

Table S2. Recyclability of catalyst POP-Ni.

Cycles	Yield (%)	Selectivity (%)
1	97.5	> 99
2	95.8	> 99
3	94.2	> 99
4	92.2	> 99
5	92.4	> 99
6	91.5	> 99

Reaction conditions**:** epichlorohydrin (5 mmol), POP-Ni (80 mg), 0.5 ml of DMF, CO_2 balloon, 24 h. The mixture was kept stirring at 80 °C for 24 h. Determined by GC-MS analysis of crude reaction samples.

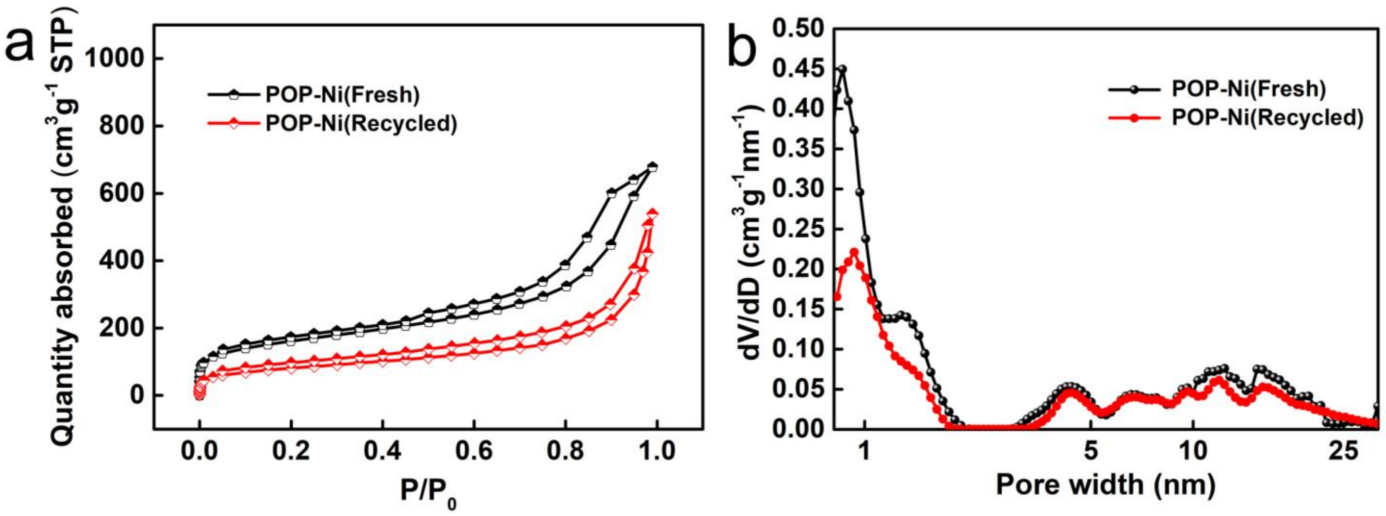

Figure S5. N_2 adsorption/desorption isotherm (a) and pore size distribution (b) of POP-Ni (fresh) and POP-Ni (recycled catalyst after the fourth run).

Disclaimer/Publisher's Note: The statements, opinions and data contained in all publications are solely those of the individual author(s) and contributor(s) and not of MDPI and/or the editor(s). MDPI and/or the editor(s) disclaim responsibility for any injury to people or property resulting from any ideas, methods, instructions or products referred to in the content.
